# Age and Gender Differences in Emotional and Behavioral Functioning Among Youth Referred to a Psychiatric Outpatient Clinic at a Public Hospital

**DOI:** 10.3390/children12060683

**Published:** 2025-05-26

**Authors:** Inbar Levkovich, Uri Yatzkar, Vered Shenaar-Golan

**Affiliations:** 1 Faculty of Education, Tel-Hai Academic College, Kiryat Shmona 1220800, Israel; 2 Director of the Child and Adolescent Psychiatry Department, Ziv Medical Center, Safed 1311502, Israel; uriy@ziv.gov.il; 3 Department of Social Work, Tel-Hai College, Kiryat Shmona 1220800, Israel; veredsh@telhai.a.il

**Keywords:** mental health, emotion regulation, depression, gender differences, adolescents, children, developmental psychopathology, anxiety, psychiatric care

## Abstract

Background: Emotional and behavioral difficulties are prevalent among children and adolescents referred for psychiatric care. This study examined how these challenges vary across age and gender, including regulatory functioning. It also explored whether emotion regulation mediates the relationship between depressive symptoms and internalizing problems and whether this mediating effect is moderated by gender and age. Methods: A cross-sectional study was conducted among 661 children and adolescents (aged 9–17 years) referred to a psychiatric outpatient clinic. Participants completed self-report questionnaires assessing depressive symptoms (MFQ-C), anxiety (SCARED-C), emotion regulation (DERS), and emotional/behavioral problems (SDQ). Statistical analyses included group comparisons and moderated mediation modeling using SPSS 27. Results: Adolescents aged 15–17 years reported significantly higher levels of depressive symptoms, emotion regulation difficulties, and anxiety-related symptoms compared to younger participants. Girls exhibited higher levels of internalizing symptoms and greater emotion regulation difficulties than boys, whereas boys showed more conduct problems. A significant gender × age interaction was found for depressive symptoms. Depressive symptoms indirectly affected internalizing problems through emotion regulation, with the strongest effect among adolescent girls. Conclusions: The findings emphasize the need for developmentally tailored assessment and intervention strategies in youth psychiatric care. Emotion regulation emerged as a central mechanism linking depressive symptoms to internalizing difficulties, particularly in older adolescent girls, supporting the design of targeted interventions to reduce emotional distress.

## 1. Introduction

Emotional and behavioral difficulties are highly prevalent among children and adolescents, with anxiety and depression consistently identified as the most common mental health disorders in this age group. Epidemiological data indicate that approximately 15% to 20% of youth are affected by anxiety disorders and 10% to 15% by depressive disorders prior to reaching adulthood [1,2]. These conditions often persist in adulthood, resulting in significant functional impairment across multiple domains [3,4]. While many studies have explored these issues in general populations, fewer have examined how they manifest in clinically referred youth, where emotional dysregulation is often more complex and multifaceted. Addressing this gap, the current study investigates how emotional, behavioral, and regulatory patterns vary by age and gender within a clinical sample. This perspective remains underexplored despite its clinical importance and potential to inform early, developmentally tailored interventions.

### 1.1. Emotion Regulation in Developmental Psychopathology

The ability to regulate emotions is increasingly recognized as a key developmental function influencing both the onset and persistence of emotional and behavioral difficulties in childhood and adolescence. Emotion regulation refers to the ways in which individuals manage the timing, intensity, and expression of their emotional experiences [5,6] Emotion regulation covers a wide spectrum of strategies, ranging from adaptive approaches such as cognitive reappraisal, acceptance, and problem-solving to maladaptive responses such as rumination, suppression, and avoidance [5]. Rather than a static set of skills, emotion regulation is now conceptualized as a dynamic system that continuously monitors and adjusts emotional states [7].

This perspective highlights the importance of understanding emotion regulation as a dynamic process rather than merely a set of skills, particularly in the context of developmental psychopathology.

Emotion regulation capacities develop across childhood and adolescence in response to neurobiological, cognitive, and social changes [8,9]. During childhood, emotional regulation is largely supported by external resources, particularly caregivers [10,11,12]. As children transition into adolescence, they increasingly rely on internal regulatory mechanisms [13], while simultaneously experiencing heightened emotional reactivity due to puberty-related hormonal changes and neurobiological development [14]. This developmental shift may lead to a temporary imbalance between emotional reactivity and regulatory control, particularly during early to mid-adolescence, when regulatory systems are still maturing and emotional responses intensify [15]. This mismatch may contribute to a temporary decrease in regulatory abilities during mid-adolescence, followed by improvements in later adolescence, potentially creating a window of vulnerability for the development of emotional disorders [16].

### 1.2. Gender Differences in Emotional and Behavioral Functioning

Research has consistently demonstrated gender differences in emotional expression, emotional difficulties, behavioral difficulties, and regulation across developmental stages, with particular divergence emerging during adolescence [17,18]. Girls typically report greater emotional intensity and awareness than boys but may also experience more problems in effectively regulating emotions, particularly negative emotions [19,20]. Recent studies have found that girls generally report higher levels of internalizing problems, such as anxiety and depression, compared to boys, with this disparity typically emerging during early adolescence and widening throughout this developmental period [11,21,22]. Conversely, boys are more likely to exhibit externalizing problems, including impulsivity, aggression, and conduct-related problems, patterns that may reflect both biological predispositions and the social reinforcement of outward behavioral expression [23].

Specifically, for depressive symptoms, a robust gender gap emerges around ages 13–15, with girls exhibiting significantly higher rates of both clinical and subclinical depression [24]. Regarding emotion regulation strategies, studies have found that girls are more likely than boys to engage in rumination, a maladaptive regulatory strategy characterized by repetitive focus on negative emotional experiences [25]. These gender differences appear to be shaped by a combination of biological, social, and contextual factors [26].

### 1.3. Emotion Regulation as a Mediator and Moderator

Recent research highlights the role of emotion regulation in shaping how psychological risks manifest. Maladaptive strategies have been found to mediate the relationship between childhood adversity and later psychopathology [27,28], indicating that regulatory difficulties may amplify vulnerability to mental health challenges. Specifically, several studies have shown that emotion regulation mediates the relationship between depressive symptoms and broader internalizing outcomes, suggesting its central role as a transdiagnostic mechanism [29,30]. These insights are particularly relevant for adolescence, a developmental window marked by increased emotional intensity and neurobiological transitions.

### 1.4. The Present Research

Despite significant advances in our understanding of emotion regulation among youth [8,9], several important gaps remain. First, while studies have established associations between emotion regulation and psychopathology, few have examined these relationships within clinical samples, particularly in outpatient psychiatric populations where a range of emotional and behavioral difficulties are present [31]. Second, research on developmental and gender-related patterns of emotion regulation has largely focused either on age or on gender, with limited exploration of their interactive effects [16]. Third, not many studies have examined the moderating role of age and gender on the mediating function of emotion regulation in clinical populations, leaving a gap in our understanding of how these mechanisms operate among more complex and symptomatic children and adolescents [32,33].

To address these gaps, the present study examined whether emotion regulation mediates the association between depressive symptoms and internalizing problems in a clinical adolescent sample, and whether this mediation varies as a function of age and gender.

Study hypotheses:

**Hypothesis** **1:** *Significant differences in levels of depressive symptoms, anxiety symptoms, emotional and behavioral difficulties, and emotion regulation difficulties will be found between different age groups*.

**Hypothesis** **2:** *Significant differences will be found between boys and girls in levels of depressive symptoms, anxiety symptoms, emotional and behavioral difficulties, and emotion regulation difficulties*.

**Hypothesis** **3:** *A significant interaction will emerge between gender and age group regarding levels of depressive symptoms, anxiety symptoms, emotional and behavioral difficulties, and emotion regulation difficulties*.

**Hypothesis** **4:** *Emotional regulation difficulties will mediate the relationship between depressive symptoms, anxiety symptoms, and emotional and behavioral difficulties, and this mediating effect will be moderated by gender and age*.

## 2. Materials and Methods

### 2.1. Participants

This cross-sectional study included 661 children and adolescents referred to an outpatient psychiatric clinic at a public hospital in northern Israel. This multidisciplinary center serves a diverse regional population and provides diagnostic and therapeutic services for a range of emotional and behavioral disorders. Upon intake, children and their families are briefly evaluated by a clinician and subsequently referred to specialized units for more in-depth assessment and treatment. Questionnaires are an integral component of the clinical admission routine. All participants and their legal guardians provided informed consent prior to inclusion. The study was approved by the Institutional Review Board (IRB) of the Ziv Medical Center in Safed, Israel (Helsinki Committee approval no. ZIV0083-19).

No imputation was applied for missing data. All analyses were conducted using available cases, and subgroup analyses were limited to participants with complete demographic and questionnaire data. Potential sources of selection bias related to clinical referral, and the geographic characteristics of the sample are addressed in the discussion section. Participants were included in the study based on clinical referral for emotional or behavioral concerns, without requiring a formal diagnostic classification.

The final sample comprised children and adolescents aged 9–17 years. Participants were classified into three developmental age groups: 9–11 (*n* = 110), 12–14 (*n* = 253), and 15–17 (*n* = 298), with the majority falling into the two older age categories. Regarding gender, 227 participants were boys and 434 were girls. Analyses were conducted using all available data, and subgroup analyses were based on complete demographic information. The present analysis explored emotional, behavioral, and regulatory patterns across age and gender groups.

The questionnaire completion process took approximately 15–20 min. Participants who encountered difficulties with reading or comprehension received assistance from trained administrative staff. Children with severe intellectual disability or insufficient proficiency in Hebrew, who were unable to complete the questionnaires independently, were not included in the study.

### 2.2. Sample Size

To ensure sufficient statistical power for the planned analyses, a priori power analysis was conducted using G*Power 3.1 to determine the required sample size [34]. The calculation was based on a multiple regression model with up to five predictors, assuming a small-to-moderate effect size (f^2^ = 0.05), conservative alpha level of α = 0.01, and high power of 0.99. The analysis indicated that a sample size of approximately 661 participants would be sufficient to detect statistically meaningful effects under these conditions. Therefore, a final sample size of 661 participants was deemed fully adequate to support the statistical robustness both of group comparisons and of multivariate regression-based analyses, including structural equation modeling and moderated mediation.

### 2.3. Measures

Emotional regulation difficulties were measured using the Difficulties in Emotion Regulation Scale (DERS) [35], a 36-item self-report questionnaire that evaluates multiple aspects of emotion dysregulation. The scale includes six subscales capturing key dimensions such as non-acceptance of emotional responses, difficulties engaging in goal-directed behavior, impulse control problems, lack of emotional awareness and clarity, and limited access to effective strategies. Participants rated each item on a 5-point Likert scale ranging from 1 (almost never) to 5 (almost always) (e.g., “When I’m upset, I have difficulty controlling my behaviors”). A total mean score was calculated, with higher scores indicating greater problems in emotion regulation. The internal consistency of the total scale in the current study was high (Cronbach’s α = 0.81).

Emotional and behavioral difficulties were assessed using the Strengths and Difficulties Questionnaire (SDQ) [36], a widely used 25-item parent-report tool. Based on Goodman et al. [37], two composite scores were computed: internalizing problems (emotional symptoms and peer problems) and externalizing problems (conduct problems and hyperactivity/inattention). Items were rated on a 3-point scale (0 = not true, 1 = somewhat true, 2 = certainly true), reflecting the child’s behavior over the past six months. Each composite score ranged from 0 to 20, with higher scores reflecting more difficulties (e.g., “Many worries, often seems worried”; “Rather solitary, tends to play alone”). Internal consistency was acceptable (Cronbach’s α = 0.78).

Depressive symptoms were assessed using the Mood and Feelings Questionnaire—Child version (MFQ-C) [38], a 33-item self-report measure for children and adolescents aged 8 to 18 years. Items describe depressive symptoms experienced in the past two weeks and are rated on a 3-point Likert scale (0 = not true, 1 = sometimes true, 2 = true) (e.g., “I felt miserable or unhappy”). The total mean score was used, with higher scores indicating more severe depressive symptoms. Internal consistency was excellent (Cronbach’s α = 0.94).

Anxiety symptoms were measured using the Screen for Child Anxiety-Related Emotional Disorders—Child Version (SCARED-C) [39], a 41-item self-report questionnaire measuring anxiety symptoms over the past three months. Items were rated on a 3-point Likert scale (0 = not true or hardly ever true, 1 = sometimes true, 2 = very true or often true) (e.g., “I am afraid to be alone at home”). The scale includes five domains: panic disorder, generalized anxiety, separation anxiety, social anxiety, and school phobia. A total mean score was calculated, with higher values reflecting more severe anxiety symptoms. Internal consistency in this study was high (Cronbach’s α = 0.92).

### 2.4. Statistical Analyses

All statistical analyses were conducted using IBM SPSS Statistics (Version 27). Before the analyses were run, the dataset was examined for missing data, outliers, and violations of normality assumptions. Descriptive statistics, including means and standard deviations, were computed for the key study variables. Reliability was evaluated using Cronbach’s alpha, with values of α ≥ 0.70 deemed acceptable. To examine group-level differences in psychological outcomes, independent samples *t*-tests (for gender) and one-way ANOVAs (for age) were performed. When applicable, Tukey HSD post-hoc tests were used to clarify significant differences between age groups. Two-way ANOVAs were conducted to test for interaction effects between gender and age groups. Effect sizes (η^2^) were reported, along with the F-values and significance levels.

A MANOVA was used to assess the combined effects of gender, age group, and their interaction on five dependent variables: anxiety symptoms, depressive symptoms, emotion regulation difficulties, internalizing problems, and externalizing problems. To address the central hypothesis, a moderated mediation model was tested using Hayes’ PROCESS macro for SPSS. This analysis evaluated whether emotion regulation difficulties mediated the association between emotional distress (depressive and anxiety symptoms) and behavioral outcomes, while also testing whether this indirect effect was moderated by gender and age. These statistical procedures were selected to align with the study’s aims, specifically to identify age- and gender-related differences and to explore the role of emotion regulation as a transdiagnostic mechanism.

## 3. Results

### 3.1. Age Differences in Emotional, Behavioral, and Regulatory Functioning

Table 1 shows the mean scores and standard deviations for all study measures across the three age groups (9–11, 12–14, and 15–17 years; N = 661). Older adolescents (15–17) reported higher levels of anxiety-related disorders, depressive symptoms, and problems in emotion regulation than younger age groups. Emotional symptoms increased slightly with age, whereas conduct problems and hyperactivity/inattention (externalizing problems) were more prominent in younger children, as reflected by the higher externalizing scores among the youngest group. Internalizing problems, which reflect emotional and peer-related symptoms, exhibited a modest increase with age. Prosocial behavior scores were highest in the youngest group (9–11).

To examine Hypothesis 1 regarding age-related differences, we classified participants into three age groups (9–11, 12–14, and 15–17 years; N = 661). A series of one-way ANOVAs were applied on the main study measures: anxiety-related symptoms (SCARED), depressive symptoms (MFQ), emotion regulation difficulties (DERS), and SDQ subscales. Older adolescents (15–17 years) reported significantly higher levels of depressive symptoms, emotion regulation difficulties, and anxiety-related symptoms than younger adolescents. Specifically, depressive symptoms exhibited significant differences (F(2) = 16.49, *p* < 0.001, η^2^ = 0.075), and emotion regulation difficulties were significantly higher in older participants (F(2) = 4.42, *p* = 0.004, η^2^ = 0.021). In contrast, externalizing behaviors (conduct problems and hyperactivity) were more prominent among younger children (F(2) = 8.13, *p* < 0.001, η^2^ = 0.038), with no significant age differences in internalizing symptoms (F = 0.88, *p* = 0.453) or in total SDQ problems (F = 1.58, *p* = 0.194) (Table 2).

**Figure 1:**
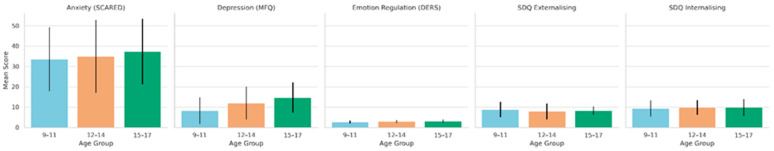
Age-group differences in depressive symptoms, externalizing behaviors, and emotion regulation.

Post-hoc Tukey tests confirmed that adolescents aged 15–17 years reported significantly higher depressive symptoms than both the 12–14 (*p* < 0.01) and 9–11 (*p* < 0.001) age groups. Emotion regulation difficulties were also significantly greater in the 15–17 group than in the youngest group (*p* < 0.05). In addition, adolescents in the 15–17 years group reported higher levels of anxiety-related symptoms, although these differences were not statistically significant. While externalizing behaviors showed a slight decline with age and internalizing symptoms increased modestly, neither of these trends reached statistical significance in the post-hoc analysis. These findings suggest that adolescence, particularly mid-to-late adolescence, is marked by increased emotional vulnerability, most notably in the domains of depression and emotion regulation (Figure 1). These results support Hypothesis 1.

### 3.2. Gender Differences in Emotional, Behavioral, and Regulatory Functioning

To examine Hypothesis 2, we conducted independent-sample *t*-tests to compare boys and girls across study measures. Girls reported significantly higher levels of anxiety (t = −5.10, *p* < 0.001), depression (t = −6.30, *p* < 0.001), and emotion regulation difficulties (t = −5.20, *p* < 0.001). They also exhibited significantly more internalizing problems on the SDQ (t = −5.75, *p* < 0.001), reflecting greater emotional and peer-related difficulties. In contrast, boys showed significantly higher externalizing problems (t = 2.41, *p* = 0.016), indicating more conduct-related and hyperactive behaviors. While girls scored slightly higher on prosocial behaviors, this difference was not statistically significant (t = −1.26, *p* = 0.209), suggesting relatively similar levels of prosocial functioning across genders. These findings suggest that girls are more prone to internal emotional distress and self-regulatory challenges, whereas boys tend to exhibit more externalized behavioral difficulties. These results support Hypothesis 2 (Table 3).

To complement the statistical significance findings, we calculated effect sizes (Cohen’s d) to estimate the magnitude of gender differences. The results indicated moderate effect sizes for depression (d = 0.515), emotion regulation difficulties (d = 0.430), and anxiety (d = 0.422), and a small effect for internalizing problems (d = 0.338). Externalizing problems showed a negligible effect size (d = −0.087), despite being statistically significant. These values suggest that the gender differences observed, particularly in depression, emotion regulation, and anxiety, are not only statistically significant but also meaningful in magnitude.

### 3.3. Gender by Age Interactions

To examine Hypothesis 3, we used two-way ANOVAs to assess the interaction effects of gender and age. A significant interaction was found for depressive symptoms (F(3) = 3.79, *p* = 0.010), indicating that gender differences in depression varied with age. A marginal interaction effect was observed for anxiety (F = 2.57, *p* = 0.053). No significant interactions emerged for emotion regulation or SDQ outcomes, although the main effects of gender were evident across variables (Table 4).

To test Hypothesis 3, we conducted a multivariate analysis of variance (MANOVA) that included five outcome variables: anxiety symptoms, depressive symptoms, emotion regulation difficulties, and SDQ internalizing and externalizing problems. The analysis revealed a significant multivariate effect for age group (Wilks’ Λ = 0.922, F(15,1590) = 3.97, *p* < 0.001), indicating age-related differences across emotional and behavioral domains. No significant multivariate effects were found for gender or gender group interactions. These findings emphasize that age, more than gender, is associated with variability across emotional and behavioral outcomes in adolescence, thus supporting Hypothesis 3 (see Table 5).

To further clarify which dependent variables contributed most to the significant multivariate effect of age group, we calculated effect sizes for each outcome variable. The results indicated that age differences were most pronounced in depressive symptoms (η^2^ = 0.088) and emotion regulation difficulties (η^2^ = 0.024), while the effects for anxiety (η^2^ = 0.008), externalizing problems (η^2^ = 0.012), and internalizing problems (η^2^ = 0.002) were notably smaller. These findings suggest that the multivariate age effect was primarily driven by differences in depression and emotion regulation.

### 3.4. Moderated Mediation Analysis

To examine Hypothesis 4, we tested a moderated mediation model to determine whether emotion regulation difficulties mediate the relationship between emotional distress (depressive and anxiety symptoms) and behavioral outcomes (internalizing and externalizing problems) and whether this indirect effect is moderated by gender and age. In the structural equation model, Paths a1 and a2, respectively, represent the associations between depressive and anxiety symptoms and problems in emotion regulation. Paths b1 and b2, respectively, assess the association between emotion regulation difficulties and internalizing and externalizing symptoms. Direct paths (c′1 and c′2) from depressive and anxiety symptoms to internalizing and externalizing outcomes are also included.

Depressive symptoms (β = 0.075, *p* < 0.001) and anxiety symptoms (β = 0.061, *p* < 0.001) were each associated with greater difficulties in emotion regulation. In turn, these difficulties predicted higher internalizing (β = 1.311, *p* < 0.001) and externalizing symptoms (β = 0.842, *p* = 0.002), even after accounting for direct effects. Both direct paths remained significant (c′1: β = 0.217, *p* < 0.001 for internalizing; c′2: β = 0.133, *p* = 0.018 for externalizing), indicating partial mediation.

The moderation analysis showed that the indirect effects varied by gender and age. For internalizing symptoms, the interaction with gender was significant (β = 0.38, *p* = 0.021), and the interaction with gender was nearly significant for externalizing symptoms (β = 0.29, *p* = 0.058). Age group also moderated the associations for internalizing (β = 0.17, *p* = 0.049) and externalizing symptoms (β = 0.14, *p* = 0.043). These findings suggest that emotion regulation difficulties mediate the impact of emotional distress on behavioral outcomes, with the indirect effect being strongest among older adolescent girls. These results support Hypothesis 4 (Table 6).

**Figure 2:**
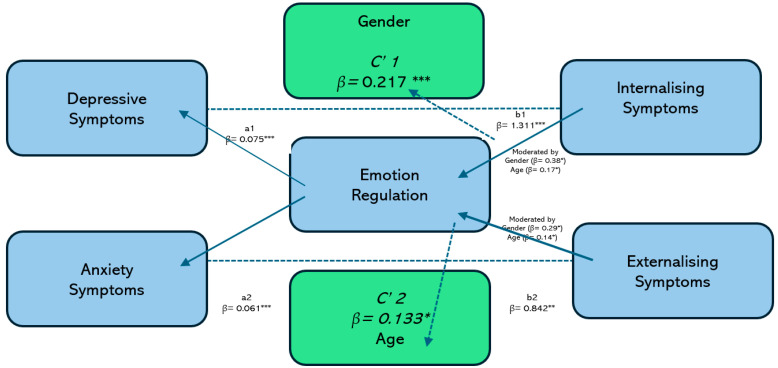
Moderated mediation model of depressive symptoms, emotion regulation, and internalizing/externalizing symptoms with moderation by gender and age. * *p* < 0.05, ** *p* < 0.01, *** *p* < 0.001.

Figure 2 shows a moderated mediation model examining whether the indirect effect of depressive symptoms on internalizing problems through emotion regulation varies as a function of gender and age. The model revealed a significant mediation pathway: depressive symptoms predicted greater emotion regulation difficulties, which, in turn, were associated with increased internalizing symptoms. Note that the strength of this indirect effect was significantly moderated by both gender and age. Specifically, the mediating effect of emotion regulation was stronger among girls than boys (β = 0.38, *p* = 0.021) and intensified with age, particularly in adolescents aged 15–17 years (β = 0.17, *p* = 0.049).

Additionally, anxiety symptoms were significantly associated with problems in emotion regulation (β = 0.065, *p* = 0.034) and internalizing symptoms (β = 1.210, *p* < 0.001). Externalizing symptoms also displayed significant associations with emotion regulation (β = 0.754, *p* = 0.012) but did not significantly moderate the indirect pathway between depressive symptoms and internalizing problems. These findings suggest that emotion regulation plays a key role in linking depression to internalizing problems. They point to the need for targeted interventions that strengthen regulation skills, particularly for older adolescent girls who are more vulnerable to internalizing difficulties.

## 4. Discussion

This study examined age- and gender-related patterns of emotional, behavioral, and regulatory functioning in children and adolescents referred to psychiatric outpatient clinics. These findings provide valuable insights into the developmental and gender-specific aspects of psychopathology in a clinical context.

Our first hypothesis predicted significant age-related differences in psychological outcomes and was largely supported by our findings. Adolescents aged 15–17 reported significantly higher levels of depressive symptoms, emotion regulation difficulties, and anxiety-related symptoms than younger participants. This pattern of increasing emotional vulnerability during mid-to-late adolescence aligns with contemporary developmental psychopathology research [23] and is also consistent with findings from other studies examining the impact of age on negative emotional reactions among Israeli populations [11,40].

The observed increase in depressive symptoms and emotion regulation problems among older adolescents provides empirical support for the “developmental mismatch” model, which emphasizes the gap between the early maturation of emotion-generative systems and the slower development of regulatory systems during adolescence [15]. This mismatch creates a window of vulnerability that may explain the heightened emotional problems observed in the 15–17 age group [41].

These age-related patterns are consistent with neurodevelopmental research showing that the limbic system matures earlier than the prefrontal cortex, creating a temporary imbalance between emotional reactivity and regulatory control during adolescence [23]. Additionally, adolescents face increasingly complex social environments and heightened expectations, which may contribute to greater emotional challenges during this developmental period [9]. These challenges underscore the importance of accurately assessing age-specific mental health needs, as supported by studies examining developmental patterns in emotion regulation abilities. Indeed, research indicates that emotion regulation capacities evolve substantially from childhood through adolescence [6] and that this developmental trajectory has significant implications for psychological well-being. Findings from both normative and clinical samples demonstrate that adolescence represents a critical period for emotional development [16], and that age-appropriate assessment and intervention are essential for effectively addressing the unique emotional needs of youth at different developmental stages [42].

Our second hypothesis regarding gender differences in emotional and behavioral functioning was supported by the data. The findings revealed distinct gender-based patterns, with girls exhibiting higher levels of internalizing symptoms and emotion regulation difficulties, and boys presenting more externalizing behaviors. These trends are consistent with prior meta-analyses and clinical studies that have highlighted gender-linked expressions of psychological distress [17,18,24] and align with broader literature examining the influence of gender on emotional responses and behavioral regulation in various developmental contexts [43].

The elevated rates of internalizing symptoms among girls are consistent with previous research demonstrating a gender gap that typically emerges between ages 13 and 15 and widens throughout adolescence [21,24,41]. This trend may be influenced by gender differences in emotional responses, perceived vulnerability, and mental health knowledge, all of which can contribute to divergent psychological outcomes during adolescence [26]. Although girls often report greater emotional awareness [19], this heightened sensitivity may increase their risk for emotional dysregulation, particularly when adaptive regulatory strategies are underdeveloped. The combination of high emotional insight and limited regulatory capacity can make girls more prone to rumination [25], a process strongly associated with anxiety and depression. Our findings support the need for prevention strategies focused on emotional regulation, tailored to gender-specific patterns of emotional processing. This is further reinforced by our results showing that, despite their emotional awareness, female adolescents report more difficulty regulating negative emotions [6], possibly due to a greater tendency to engage in rumination [25,28].

The gender differences we observed likely stem from multiple factors. Biological hormonal changes during puberty may contribute to gender-differentiated patterns of emotional reactivity and regulation [44]. Socialization practices and cultural expectations also differentially affect boys’ and girls’ emotional expression and regulatory strategies, with girls often encouraged to attend to their emotions while boys are directed toward suppressing vulnerability [45].

Our third hypothesis predicted that gender differences would vary by age group. This was partially supported by our findings that pointed to a significant interaction for depressive symptoms, indicating that gender differences in depression vary throughout development. This interaction effect aligns with research documenting the emergence of gender disparities in depression during early adolescence [24].

The significant interaction observed for depressive symptoms suggests that gender differences in emotional vulnerability change and intensify throughout adolescence. This developmental shift aligns with longitudinal research showing that depressive symptoms tend to emerge and become more pronounced in girls during mid-adolescence [24]. The expanding gender gap may be influenced by a combination of biological, social, and cognitive factors, including puberty-related hormonal changes, gendered social expectations [45], and increased demands for emotional self-regulation in socially complex environments [9].

Our MANOVA results revealed a significant multivariate effect for age group but not for gender or gender × age group interactions across the combined outcomes. This finding emphasizes the primacy of developmental stage, rather than gender alone, in shaping the constellation of emotional and behavioral symptoms during adolescence [30]. Qualitative studies examining adolescents’ experiences in challenging family situations have shown similar patterns in which the interaction of age and gender plays a role in the experience of emotional symptoms and coping strategies [46].

Our fourth hypothesis proposed that the indirect relationship between depressive symptoms and internalizing problems, mediated by emotion regulation, would be moderated by both gender and age. The results supported this prediction: emotion regulation difficulties significantly mediated the association between depressive symptoms and internalizing symptoms, with the strongest effects observed among older adolescent girls. These findings are consistent with prior mediation research indicating that difficulties in emotion regulation account for a substantial part of the link between depressive symptoms and broader internalizing outcomes [29]. They also align with transdiagnostic models of psychopathology that identify emotion regulation as a core mechanism of vulnerability across mental health conditions [13,28]. The pronounced mediation effect in girls reflects documented gender differences in the developmental course of emotion regulation [47] and may stem from greater use of maladaptive strategies such as rumination, which is closely associated with anxiety and depression [25]. This gender-specific pathway was further supported by a significant interaction between emotion regulation and gender in predicting internalizing symptoms. As shown in prior work by Gonçalves et al. [47], boys and girls follow distinct developmental trajectories in emotion regulation. The current findings underscore the importance of targeting emotion regulation in interventions, particularly for older adolescent girls at elevated risk for co-occurring depressive and anxiety disorders [48,49].

### 4.1. Clinical Implications

Our findings have several important clinical implications. First, they highlight the need for age-sensitive assessment and intervention approaches in youth psychiatric services. The increased emotional vulnerability observed in older adolescents, particularly depressive symptoms and emotion regulation difficulties, suggests that clinicians should pay special attention to these domains when working with adolescents aged 15–17 [42].

Second, the significant gender differences observed across multiple domains underscore the importance of gender-sensitive approaches to assessment and treatment. The finding that girls report higher levels of internalizing symptoms and emotional regulation problems suggests that interventions targeting these domains may be particularly beneficial for female adolescents [17].

Third, the moderated mediation findings provide empirical support for targeting emotion regulation as a transdiagnostic treatment mechanism. Interventions that enhance adaptive emotion regulation strategies may be particularly beneficial in reducing internalizing symptoms, especially among older adolescent girls [48]. Therapeutic approaches, such as emotion regulation therapy, dialectical behavior therapy, and mindfulness-based interventions, that explicitly address regulatory skills may be especially valuable in this context [49].

Fourth, our findings suggest that clinical assessments should routinely evaluate emotion regulation capacity alongside symptom measures. The DERS provides a brief yet comprehensive assessment of regulatory problems that can inform case conceptualization and treatment planning [42]. Identifying youth with severe emotion regulation problems may help prioritize interventions for those at the highest risk of persistent psychopathology. Understanding the factors influencing professionals’ responses to adolescents provides additional insights into ways to improve the identification and treatment of adolescents experiencing emotion regulation difficulties [50].

Finally, these findings also carry implications for early identification and prevention efforts beyond clinical settings. School-based interventions that focus on strengthening emotion regulation skills, such as SEL (social and emotional learning) programs or mindfulness training, may serve as effective preventive strategies, particularly when implemented before the peak vulnerability of mid-adolescence. Universal and targeted programs that build adaptive coping strategies could help mitigate the onset of internalizing symptoms in at-risk youth. Collaboration between mental health professionals and educators is essential for integrating these approaches into school curricula and for creating supportive environments that promote emotional resilience across developmental stages.

### 4.2. Limitations

Although the study yielded meaningful results, several limitations must be acknowledged. First, its cross-sectional nature restricts the ability to draw causal conclusions between variables. Longitudinal approaches are necessary to determine directionality and developmental trends. Second, the exclusive use of self-report instruments may have introduced biases related to social desirability and participants’ limited self-insight. Future studies should incorporate multi-informant approaches and behavioral measures of emotion regulation. Third, although our sample was substantial, all participants were from a single psychiatric center in northern Israel, potentially limiting the generalizability to more diverse populations. Cross-cultural validation would strengthen the external validity of these findings. In addition, important sociodemographic factors, such as participants’ residential setting, family composition, academic functioning, or history of hospitalization and comorbid psychiatric conditions were not assessed and may help explain observed age and gender differences. These variables could offer meaningful context for interpretation and should be included in future research. Moreover, cultural factors specific to the Israeli context, such as prevailing norms around emotional expression and gender roles, may have influenced the patterns of reported symptoms. Finally, for the purposes of this research, we grouped participants based on presentations of emotional and behavioral difficulties without differentiating among specific diagnoses, which may have obscured important diagnostic differences in emotion regulation patterns.

## 5. Conclusions

This study provides significant evidence of age- and gender-related differences in emotional, behavioral, and regulatory functioning among children and adolescents referred to for psychiatric care. Our findings highlight the increased emotional vulnerability of adolescents aged 15–17, particularly girls, and reveal the pivotal role of emotion regulation as a psychological mechanism linking depression to internalizing problems. These insights emphasize the importance of age- and gender-sensitive assessment practices and support the development of targeted interventions, such as school-based programs and early screening initiatives, aimed at improving emotion regulation and reducing internalizing symptoms in at-risk youth. The complex interplay between developmental processes, gender, emotion regulation, and psychopathology revealed in this study underscores the importance of adopting a nuanced and developmentally sensitive approach in clinical practice for youth. While the study contributes valuable knowledge, its generalizability is limited by the single-site sample and reliance on self-report measures. Future research should adopt longitudinal and multi-informant approaches to better clarify causal pathways and extend the findings to broader populations. It would also be beneficial to examine additional contextual variables, such as social support and family dynamics, which may further explain age- and gender-related differences in symptom expression.

## Figures and Tables

**Table 1:** Questionnaire means (SD) by age group (*n* = 661).

	9–11 (*n* = 110)	12–14 (*n* = 253)	15–17 (*n* = 298)
Anxiety-related disorders	33.62 (15.73)	34.96 (18.03)	37.35 (16.17)
Depressive symptoms	8.26 (6.50)	12.02 (7.99)	14.77 (7.41)
Difficulties in emotion regulation	2.80 (0.69)	2.96 (0.81)	3.13 (0.77)
SDQ—externalizing	8.90 (3.71)	7.89 (3.89)	8.22 (2.11)
SDQ—internalizing	9.35 (3.95)	9.83 (3.61)	9.88 (4.11)

Note: SD = standard deviation; SDQ = Strengths and Difficulties Questionnaire.

**Table 2:** ANOVA results for age group differences in questionnaire scores (*n* = 661).

Variable	F	df	*p*	η^2^
Anxiety-related disorders	1.12	2	0.339	0.006
Depressive symptoms (MFQ)	16.49	2	<0.001	0.075
SDQ—internalizing	0.88	2	0.453	0.004
SDQ—externalizing	8.13	2	<0.001	0.038
Emotion regulation (DERS)	4.42	2	0.004	0.021

Note: η^2^ = eta squared; *p*-values reflect the significance of one-way ANOVA tests.

**Table 3:** Descriptive statistics and *t*-test results by gender (boys vs. girls) (*n* = 661).

	Boys: M (SD)	Girls: M (SD)	*t*-Value	*p*-Value
Anxiety-related disorders	31.41 (16.72)	38.42 (16.57)	−5.1	<0.001
Depressive symptoms	10.15 (7.39)	14.06 (7.69)	−6.3	<0.001
Difficulties in emotion regulation	2.80 (0.72)	3.13 (0.79)	−5.2	<0.001
SDQ—internalizing	8.77 (3.98)	10.10 (3.92)	−5.57	<0.001
SDQ—externalizing	8.56 (3.74)	8.23 (3.82)	2.41	0.017

Note: M = mean, SD = standard deviation. Independent sample *t*-tests were used to assess gender differences. Higher scores indicate greater problems for all scales except Prosocial Behavior, where higher scores reflect more prosocial functioning. *p*-values were based on independent sample *t*-tests. Gender groups: boys (*n* = 227), girls (*n* = 434).

**Table 4:** Two-way ANOVA results: gender × age group (*n* = 661).

Variable	F (Gender)	*p* (Gender)	F (Age)	*p* (Age)	F (Interaction)	*p* (Interaction)
Anxiety-related disorders	23.88	<0.001	0.84	0.473	2.57	0.053
Depressive symptoms	24.02	<0.001	10.99	<0.001	3.79	0.010
Total problems	5.13	0.024	2.38	0.069	1.65	0.176
Internalizing symptoms	15.22	<0.001	0.25	0.860	1.54	0.202
Externalizing symptoms	0.04	0.846	7.50	<0.001	2.02	0.109
Emotion regulation difficulties	21.11	<0.001	2.20	0.087	1.85	0.137

Note: F = F-value from ANOVA; *p* = significance level. “Interaction” refers to the interaction effect of gender × age group.

**Table 5:** MANOVA results—effects of gender, age group, and their interaction (*n* = 661).

Effect	Wilks’ Lambda	F	df1	df2	*p*-Value
Gender	0.995	0.61	5	602	0.660
Age group	0.922	3.97	15	1590	<0.001
Gender × age group	0.973	1.39	15	1590	0.158

Note: A multivariate analysis of variance (MANOVA) was conducted using Wilks’ lambda to test multivariate effects across five variables: anxiety symptoms, depressive symptoms, emotion regulation difficulties, and SDQ internalizing and externalizing problems.

**Table 6:** Moderated mediation model (*n* = 661).

Predictor	Path	Outcome Variable	β	*p*-Value
Depressive symptoms	a1	Emotion regulation (DERS)	0.075	<0.001
Anxiety symptoms	a2	Emotion regulation	0.061	<0.001
Emotion regulation	b1	Internalizing symptoms	1.311	<0.001
Emotion regulation	b2	Externalizing symptoms	0.842	0.002
Depressive symptoms	c′1	Internalizing symptoms	0.217	<0.001
Anxiety symptoms	c′2	Externalizing symptoms	0.133	0.018
DERS × gender	m1	Internalizing symptoms	0.38	0.021
DERS × gender	m2	Externalizing symptoms	0.29	0.058
DERS × age group	m3	Internalizing symptoms	0.17	0.049
DERS × age group	m4	Externalizing symptoms	0.14	0.043

Note: Path a represents the predictors of the mediator (problems in emotion regulation). Path b represents predictors of the outcome variable (internalizing symptoms).

## Data Availability

The datasets presented in this article are not readily available because of privacy and sensitivity issues. Requests to access the datasets should be directed to the lead author.
